# Visibility-aware Fisher information optimization for optimal fiducial marker deployment in complex indoor environments

**DOI:** 10.1038/s41598-026-55054-2

**Published:** 2026-06-19

**Authors:** Banafshe Akbarinia, Meor Faisal Zulkifli, Bushroa Abd Razak, Mohd Ridha Muhamad, Hamed Shahmohamadi Ousaloo, Norrima Mokhtar

**Affiliations:** 1 Department of Mechanical Engineering, Faculty of Engineering, University of Malaya, 50603 Kuala Lumpur, Malaysia; 2 Centre of Research in Industry 4.0, University of Malaya, 50603 Kuala Lumpur, Malaysia; 3 Electrical & Robotics Engineering Department, School of Engineering, Monash University Malaysia, 47500 Bandar Sunway, Selangor Malaysia; 4 Department of Electrical Engineering, Faculty of Engineering, University of Malaya, 50603 Kuala Lumpur, Malaysia

**Keywords:** Fiducial markers, Optimal marker placement, Fisher information, Visual localization, Visibility aware selection, Matterport point cloud, Engineering, Mathematics and computing

## Abstract

Fiducial markers are fundamental tools for visual localization, calibration, and augmented reality; however, the precision of these systems is fundamentally constrained by the spatial configuration of the markers. We introduce a principled pipeline for optimal marker deployment in indoor scenes. The method (i) extracts wall-like candidate locations from dense 3D reconstructions, (ii) computes a Fisher Information Matrix (FIM) for each candidate by analytically aggregating the Jacobians of projected marker corners over a set of camera poses, and (iii) selects a compact subset of markers via an information-aware optimization strategy. Given a Matterport-style point cloud, the system automatically proposes marker poses on planar surfaces at user-specified heights. The proposed framework was validated through both simulation and real-world indoor experiments and compared against random, uniform, and optimization-based baseline strategies.

## Introduction

Fiducial markers provide geometric constraints for visual localization and camera calibration and are widely used in robotics^1^, AR/VR^2,3^, and metrology^4^. Although detection of planar markers such as ArUco^5^, AprilTag^6^ is well established, the problem of where these markers should be placed within an environment remains largely underexplored and is often addressed using heuristic practices.

Marker placement fundamentally influences the observability and conditioning of the pose-estimation problem. Poorly distributed markers may lead to geometric degeneracies, weak measurement sensitivity, and increased uncertainty propagation, particularly under limited viewpoints and occlusions^7,8^. Conversely, well-distributed markers improve geometric diversity and strengthen the sensitivity of image measurements to pose perturbations, thereby reducing estimation uncertainty. From an estimation-theoretic perspective, fiducial marker deployment can therefore be interpreted as an experimental design problem, where the spatial configuration of markers determines the amount of information available for camera pose estimation. This provides the scientific motivation for formulating marker placement using the Fisher Information Matrix (FIM) and evaluating achievable localization accuracy through the Cramér–Rao Lower Bound (CRLB).

Despite this importance, marker deployment in practice is commonly based on convenience, uniform spacing, or visual intuition. Such approaches neglect the underlying information structure of the observation model and may fail in cluttered or complex indoor scenes^1^. A principled framework is therefore needed to guide deployment decisions based on measurable estimation-theoretic criteria.

This paper formulates marker placement as a FIM optimization problem derived from pixel-level reprojection measurements.

Maximizing the log-determinant of the FIM minimizes the CRLB on 6-DOF pose estimation, providing a physically interpretable and prior-independent measure of achievable accuracy. This formulation explicitly incorporates marker geometry, camera intrinsics, visibility constraints, and occlusion effects.

**Fig. 1:**
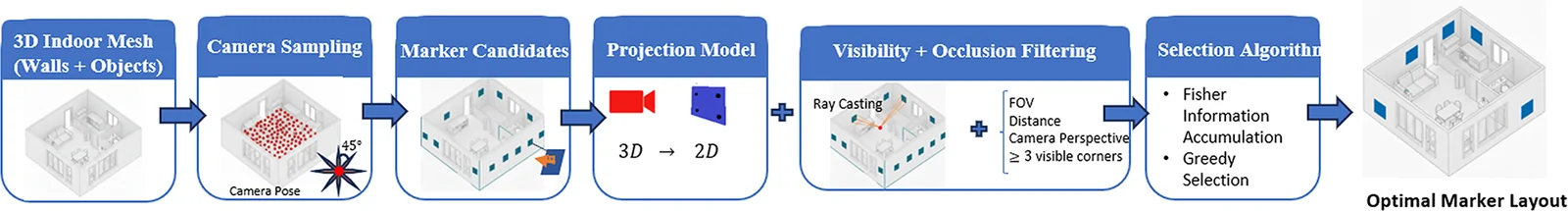
Overview of the proposed Fisher-information–guided fiducial marker placement pipeline. Given a 3D indoor mesh, candidate camera poses and marker locations are generated. Marker visibility is evaluated through projection and occlusion analysis, and their information contribution is quantified via the Fisher Information Matrix. A greedy log-determinant maximization selects the final marker subset that maximizes pose observability.

The proposed pipeline automatically extracts feasible marker locations from dense 3D reconstructions, samples valid camera viewpoints through voxelized free-space analysis, evaluates visibility via mesh-based ray casting, and computes per-marker information contributions using analytic Jacobians. A greedy D-optimal selection strategy then identifies a compact subset of markers that maximizes overall observability. An overview of the proposed framework is shown in Fig. 1.

The main contributions of this study are summarized as follows:

1. *FIM-driven formulation for marker placement*

We cast fiducial-marker placement as an optimization problem based on the Fisher Information Matrix derived from pixel-level reprojection measurements, establishing a direct link between marker geometry and pose-estimation accuracy.

(2) *CRLB-based observability criterion*

By maximizing the log-determinant of the FIM, the proposed method minimizes the Cramér–Rao Lower Bound for 6-DOF pose estimation, providing a prior-independent and physically interpretable accuracy metric.

(3) *Visibility- and occlusion-aware information modeling*

The framework explicitly incorporates field-of-view limits, depth validity, surface orientation, and mesh-based occlusion into the information model to ensure realistic measurement conditions.

(4) *End-to-end design pipeline from 3D reconstructions*

We develop a complete pipeline that extracts feasible marker locations from dense meshes, samples valid camera viewpoints via voxelized free space, and evaluates per-marker information contributions using analytic Jacobians.

(5) *Comparison with entropy-greedy baseline and quantitative validation*

The proposed visibility-aware FIM optimization is quantitatively compared against a Huang-inspired entropy-greedy (HIEG) baseline, as well as uniform and random deployment strategies, through Monte Carlo simulations and real-world laboratory experiments. Results demonstrate improved localization accuracy.

## Related work

Fiducial markers have long served as reliable geometric anchors in visual localization, camera calibration, and pose estimation systems. By encoding planar coordinate patterns that can be robustly detected in images, they enable accurate six-degree-of-freedom (6-DOF) pose estimation under diverse operating conditions. Widely adopted marker families such as ArUco^5^, AprilTag^6^, and Stag^3^ have emphasized robustness, computational efficiency, and pose stability across varying illumination, scale, and perspective distortions. Consequently, fiducial markers have become essential components in robotics^1,9,10^, augmented/virtual reality (AR/VR)^2,3^, industrial metrology^4^, and autonomous navigation systems^11,12^, where precise and repeatable spatial estimation is required.

While substantial advances have been achieved in marker design, coding strategies, and detection algorithms^5,9,13–15^, comparatively less attention has been devoted to the equally critical problem of marker deployment. In many real-world applications, markers are still arranged manually, uniformly distributed, or positioned based on operator intuition. Such approaches rarely account for environmental geometry, visibility constraints, or the conditioning of the underlying estimation process^1,7,8^. As a result, marker layouts that appear intuitively reasonable may yield suboptimal localization performance, particularly in cluttered indoor environments where occlusions and restricted viewpoints are common.

Early studies addressing marker placement largely relied on heuristic or metaheuristic optimization strategies. Allen et al. employed metaheuristic search to optimize directional marker placement for mobile robot navigation^16^, while Mantha and García de Soto^17^ investigated spacing and layout strategies through ROS/Gazebo simulation. In medical imaging, simulation-based optimization has been used to minimize target registration error through fiducial arrangement design^7^. These studies demonstrated that placement significantly affects localization quality; however, their optimization objectives were often task-specific, empirically defined, or based on ad hoc scoring functions without a rigorous connection to estimator precision.

Subsequent research introduced environment-aware placement strategies that incorporated scene geometry and viewpoint coverage. Representative efforts include maximizing marker visibility across camera networks^18^, designing layouts that avoid degenerate viewing configurations^19^, and improving joint observability in multi-camera systems^8^. More recent studies on indoor localization have further analyzed the influence of marker orientation, spacing, and distance on positioning accuracy in structured environments^20^. Similarly, García-Ruiz et al.^8^ demonstrated that marker arrangement strongly influences reprojection consistency and positioning robustness in expansive indoor spaces. While these methods improve deployment practicality, their evaluation criteria remain largely heuristic or simulation-driven, limiting generalizability and theoretical interpretability.

To establish more principled optimization criteria, recent work has increasingly adopted information-theoretic formulations. Mutual information (MI) and entropy-based methods aim to maximize uncertainty reduction by selecting configurations that provide the highest expected information gain. For example, Caron et al.^21^ demonstrated the effectiveness of MI as an optimization objective for robust pose estimation under uncertain visual conditions, while hybrid frameworks combining Bayesian Fisher information and mutual information have demonstrated effectiveness in balancing global uncertainty reduction with local estimator sensitivity^22^. These approaches offer strong probabilistic foundations; however, they often require extensive sampling, posterior approximation, or high-dimensional probabilistic modeling, which can become computationally demanding for large-scale indoor deployment problems.

In parallel, estimation-theoretic approaches based on the FIM have gained prominence as a computationally efficient and statistically grounded alternative. The FIM quantifies the sensitivity of image measurements to pose parameters and provides a lower bound on achievable estimation variance through the CRLB^23^. FIM-based optimization, equivalently minimizing the CRLB, has been applied to sensor placement^24,25^, multi-camera calibration^26–28^, and visual localization system design^29,30^. Recent advances have extended FIM frameworks through modified information matrices, effective independence criteria, and incorporation of full error covariance structures, thereby improving robustness and applicability in complex sensing environments^31^, and extended FIM formulations to account for correlated uncertainty structures Compared with entropy-based formulations^32^. Unlike entropy-based placement methods that optimize generalized uncertainty reduction without explicitly modeling geometric visibility constraints, the proposed framework incorporates pixel-level reprojection sensitivity together with line-of-sight visibility and occlusion into the optimization process.

Beyond FIM, observability-driven frameworks have also emerged as an alternative theoretical basis for placement optimization. Approaches based on empirical observability Gramians assess how effectively system states can be inferred under different measurement configurations, particularly in nonlinear systems^33^. These methods provide deeper insight into system dynamics and measurement sensitivity, but they often incur substantial computational cost and may be difficult to scale in large indoor environments.

Another related stream of work optimizes marker placement through direct minimization of localization or reprojection error^18^. These approaches prioritize task-level performance metrics and often rely on simulation or experimental measurement to identify effective configurations. While highly relevant for application-specific deployment, such methods are typically post hoc and require repeated evaluation under predefined scenarios. By contrast, estimation-theoretic criteria enable predictive assessment of configuration quality before physical deployment, reducing reliance on exhaustive empirical testing and improving transferability across environments.

Building upon our earlier discussions, existing marker deployment approaches include heuristic placement strategies, entropy- and mutual-information-based optimization, localization-error-minimization methods, and estimation-theoretic formulations based on observability or Fisher information. While these methods improve deployment quality, many either rely on computationally intensive probabilistic modeling, empirical evaluation metrics, or simplified sensing assumptions. To the best of our knowledge, this work is among the first to formulate fiducial marker deployment directly as a visibility-aware FIM optimization problem derived from pixel-level reprojection sensitivity. The proposed framework addresses these limitations by integrating geometric observability together with realistic line-of-sight and occlusion constraints into the marker placement process.

## Methodology

### Problem formulation

Consider a 3D environment with candidate marker positions and associated surface normal ${n}_{i}$, centered at

1 $$\:\chi\:={\left\{{\varkappa\:}_{i}\right\}}_{i=1}^{N}$$

where *N* is the all-possible marker positions in the environment. A camera pose *Θ* is parameterized by a 6-vector observing a set of planar fiducial markers placed in a 3-D environment.

Each ${\Theta}_{j}\in {\mathbb{R}}^{6}$, parametrized as $\Theta ={\left[{\phi }^{T},{t}^{T}\right]}^{T}={[\varphi ,\theta ,\psi , x,y,z]}^{T}$ where *t* is translation and *ϕ* is a minimal rotation vector (e.g. axis-angle / Rodrigues) denote the camera pose parameters. The world to camera rotation matrix is *R(ϕ )* ($R\left(\varphi ,\theta ,\psi \right)={R}_{z}\left(\psi \right){R}_{y}\left(\theta \right){R}_{x}(\varphi )$). Set of sampled camera poses $\complement ={\left\{\left({R}_{c},{ t}_{c}\right)\right\}}_{c=1}^{M}$ used to evaluate candidate informativeness.

### Measurement model

Marker *i* has known geometry (square marker with 4 corner 3D points given pose ${p}_{i}$​ on surface ${\left\{{p}_{i,k}\right\}}_{k=1}^{4}$ computed from center ${\varkappa}_{i}$, normal ${n}_{i}$ and size *s**.* With camera intrinsics matrix $$K = \left[ {\begin{array}{*{20}c} {f_{x} } & 0 & {c_{x} } \\ 0 & {f_{y} } & {c_{y} } \\ 0 & 0 & 1 \\ \end{array} } \right]$$ (The parameters ${f}_{x}\, and\, {f}_{y}$ denote the focal lengths, and ${c}_{x}, {c}_{y}$ are the principal point coordinates of the camera intrinsic matrix K) The deterministic projection of the marker points is:

2 $$h\left( {p_{i,k} } \right) = \frac{1}{Z}\left[ {\begin{array}{*{20}c} {f_{x} X + c_{x} Z} \\ {f_{y} Y + c_{y} Z} \\ \end{array} } \right] = \left[ {\begin{array}{*{20}c} u \\ v \\ \end{array} } \right]$$

where $\left[ {X,Y,Z} \right]^{T}$ = $R\left( {\varphi ,\theta ,\psi } \right)\left( {p_{i,k} - t} \right)$ = $R\left( {p - t} \right)$ from world coordinates to camera coordinates, constructed from roll *φ*, pitch *θ*, and yaw *ψ* angles.

**Fig. 2:**
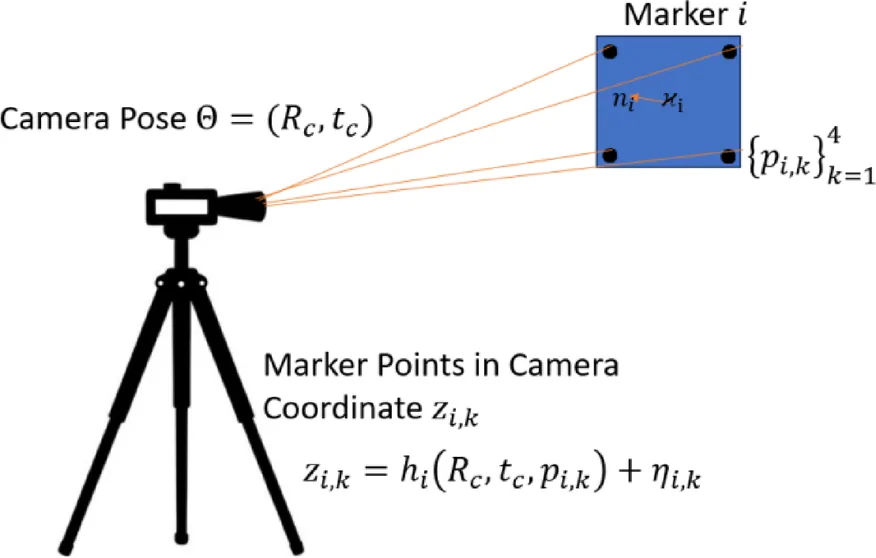
Projection model for a planar fiducial marker.

Observed pixel measurement of marker corners *k* when seen by camera *i* is:

3 $$z_{i,k} = h_{i} \left( {R_{c} ,t_{c} ,p_{i,k} } \right) + \eta_{i,k}$$

where $h_{i}$ is the nonlinear projection function determined by the pinhole camera model. For camera observing marker *i*, the *2D* pixel observations are $z_{i,k} \in {\mathbb{R}}^{2M}$ (for *M = 4* corners for each marker). $\eta_{i,k}$ is zero-mean Gaussian noise with covariance $\sum_{i,k}$ $\left( {\eta_{i,k} \sim {\mathcal{N}}\left( {0,\sum_{i,k} } \right) } \right)$. In many models we can consider identical isotropic pixel noise $\sum_{i,k} = \sigma^{2} I$ for all corners. The geometric relationship between the 6-DOF camera pose and the projected 2D coordinates of the fiducial marker corners is illustrated in Fig. 2, which defines the measurement model used to derive the Fisher Information Matrix. In Fig. 2, the 3D coordinates of the four corners $\left\{ {p_{i,k} } \right\}_{k = 1}^{4}$ are transformed into the camera coordinate system based on the camera pose ${\Theta } = (R_{c} ,t_{c} ).{ }$ The observed pixel measurement $z_{i,k}$ is a function of the nonlinear projection $h_{i}$ and additive Gaussian noise $\eta_{i,k}$, forming the basis for the information-theoretic optimization.

### Visibility

A binary visibility indicator is assigned to each marker corner based on (i) perspective (point in front of camera), (ii) image bounds, and (iii) line-of-sight (raycast occlusion). We allow a probabilistic visibility that models partial corner occlusion or detection failure. Later we incorporate visibility into expected FIM.

4 $${\mathcal{P}}_{i,c,k} \in \left[ {0,1} \right]$$

### Fisher information computation

Each marker provides constraints on the 6-DOF camera pose through its projected corners. The per-marker contribution is accumulated across cameras to build FIM. For a fixed camera pose $(R_{c} ,t_{c} )$ and a candidate marker *i*, stack the 2D reprojections of the four corners into a vector $z_{i,c} = \left[ {z_{i,c,1}^{T} , \ldots , z_{i,c,4}^{T} } \right] \in {\mathbb{R}}^{8}$, let the stacked noise covariance be $\sum_{i,c} = diag\left( {{\Sigma}_{i,c,1} , \ldots , {\Sigma}_{i,c,4} } \right) \in {\mathbb{R}}^{8 \times 8}$.

Assuming independent Gaussian noise,

5 $$p_{i,c} \left( {\left. z \right|{\Theta }} \right) \propto {\mathrm{exp}}\left( { - \frac{1}{2}\left( {h_{i,c} \left( p \right) - z_{i,c} } \right)^{T} {\Sigma}_{i,c}^{ - 1} \left( {h_{i,c} \left( p \right) - z_{i,c} } \right)} \right.$$

The negative log-likelihood is quadratic locally near the maximum-likelihood estimate^23^; the FIM for *θ* is:

6 $$F_{i,c} \left( {\Theta } \right) = {\mathbb{E}}\left[ {\nabla_{{\Theta }} \ell \nabla_{{\Theta }} \ell^{T} } \right] = J_{i,c}^{T} {\Sigma}_{i,c}^{ - 1} J_{i,c} \in {\mathbb{R}}^{6 \times 6}$$

Intuitively, each visible marker contributes pose information proportional to how sensitively its projected corners move under small pose perturbations.

where $J_{i,c} = \frac{{\partial h_{i,c} \left( {R_{c} ,t_{c} ,p_{i,k} } \right)}}{{\partial {\Theta }}} \in {\mathbb{R}}^{8 \times 6}$ is the Jacobian of the stacked corner reprojections with reference to the 6-DOF pose (The detailed analytical derivation is provided in the Appendix. This is standard Fisher/CRLB calculus; the CRLB states^34^:

7 $$Cov\left( {{\hat{\Theta }}} \right) \ge F\left( {\Theta } \right)^{ - 1}$$

Aggregate across all sampled cameras to obtain the per-marker FIM

8 $$F_{i} = \mathop \sum \limits_{c = 1}^{M} {\mathcal{P}}_{i,c} F_{i,c}$$

where ${\mathcal{P}}_{i,c}$ is the average corner visibility (${\mathcal{P}}_{i,c} = \frac{1}{4}\mathop \sum \limits_{k = 1}^{4} {\mathcal{P}}_{i,c,k}$ and ${\mathcal{P}}_{i,c,k} \in \left[ {0,1} \right]$).

Given a subset $S \subseteq \left\{ {1, \ldots , N} \right\}$ of size *k*, the goal is to minimize pose estimation uncertainty. Under an information-theoretic framework, this task is reformulated as the maximization of the FIM determinant. For the selected set *S*, the total information is represented by

9 $$F\left( S \right) = \mathop \sum \limits_{i \in S} F_{i}$$

The optimization objective is thus to select a subset *S* that maximizes the log-determinant of the global FIM:

10 $$\:{S}^{*}=arg\underset{S:\:\left|S\right|=K}{\mathrm{max}}logdet\left(F\left(S\right)\right)$$

### Selection algorithms

To solve the optimization problem, we employ a greedy selection strategy. The algorithm iteratively selects the marker *i* that maximizes the marginal information gain:

11 $$\vartriangle_{i} = logdet\left( {F_{current} + \widetilde{{F_{i} }}} \right) - logdet\left( {F_{current} } \right)$$

Here, $F_{current}$ is the cumulative FIM of previously selected markers, and $\widetilde{{F_{i} }}$ is the contribution of the candidate marker. This process repeats until the cardinality constraint $\left|S\right|=k$ is met.

### Visibility-aware greedy marker selection algorithm

The overall visibility-aware FIM optimization procedure for fiducial marker deployment is summarized in Algorithm 1.

**Algorithm 1:**
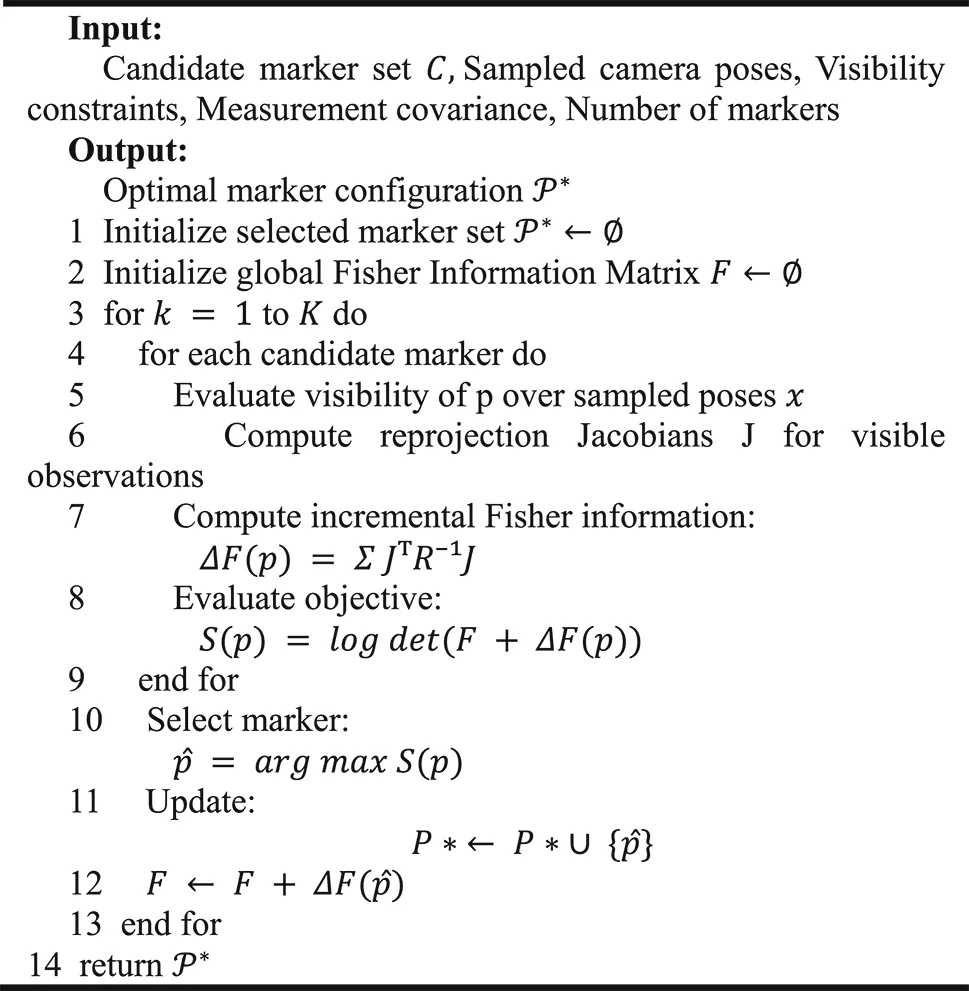
Visibility-aware FIM marker deployment.

## Experimental setup

### Dataset and simulation setup

We evaluate the proposed fiducial-marker placement strategy using Matterport 3D scenes (Scenes #1–3) which provide realistic indoor environments with detailed wall and object geometries. Candidate marker positions are sampled at 0.5 m intervals along visible wall surfaces, and marker size is fixed at 15 cm. Cameras are positioned at a height of 1.5 m, with 8 orientations per location (45° intervals) to simulate multi-view observations. Camera intrinsics are ${f}_{x}={f}_{y}=800$, ${c}_{x}=320, {c}_{y}=240$, and the image resolution is *640× 480*.

A maximum camera-to-marker distance of 10 m is enforced, and markers are considered visible if at least 3 of 4 corners are detectable. Visibility is further constrained by field-of-view limits and occlusions, which are evaluated via ray casting in the 3D scene.

Monte Carlo simulations are performed to assess the robustness of placement strategies (50 trials). Each trial randomly samples camera poses within a predefined observation volume, projecting all marker corners onto the image plane. Observations are corrupted with zero-mean Gaussian noise.

12 $$\eta_{ij} \sim {\mathcal{N}}\left( {0,{\Sigma}_{ij} } \right), \quad {\Sigma } = \sigma_{pix}^{2} I_{8 \times 8}$$

where ${\sigma}_{pix}=1.5$ pixels and *i,j* index the corners of each marker.

### Physical laboratory testbed

Empirical validation was performed in a structured laboratory environment measuring 9.80 *×* 5.72 *×* 3.0 m (Scene #4). The facility was configured with internal partitions and stationary obstacles to replicate complex line-of-sight constraints and realistic indoor occlusions.

Optical data was acquired using a Teledyne FLIR BFS-U3-63S4M-C monochrome industrial camera, featuring a resolution of 3072 *×* 2048 px, 2.4 *μ m* pixel pitch. The camera’s intrinsic parameters *K* and radial/tangential distortion coefficients *D* were pre-calibrated as follows:

$$K = \left[ {\begin{array}{*{20}c} {2528.62} & 0 & {1522.82} \\ 0 & {2543.67} & {1079.42} \\ 0 & 0 & 1 \\ \end{array} } \right],$$

$$D = \left[ {\begin{array}{*{20}c} { - 0.048} & { - 0.120} & {\begin{array}{*{20}c} {0.008} & {\begin{array}{*{20}c} { - 0.002} & {0.523} \\ \end{array} } \\ \end{array} } \\ \end{array} } \right]$$

The testbed utilized AprilTag 36h11 fiducial markers with a physical size of 13 *×* 13 cm. To maintain a consistent geometric plane, both the markers and the camera optical center were positioned at a fixed height of 1.0 m. Simulation parameters mirrored the physical environment, assuming a ${62}^{^\circ }$ FOV and *σ =1* pixel measurement noise for FIM computation. Ground-truth (GT) poses were established via manual grid measurements at headings of ${0}^{^\circ }, {90}^{^\circ }, {180}^{^\circ }, {270}^{^\circ }$ Given the inherent uncertainty in manual GT acquisition, these experiments primarily serve to validate the relative performance of the proposed deployment methods under realistic constraints.

The primary objective of the laboratory experiment is to validate relative deployment performance trends under practical sensing conditions rather than to establish sub-centimeter metrology accuracy. As ground-truth measurements were obtained manually, the reference data contains non-negligible measurement uncertainty, particularly regarding orientation estimation. Consequently, the analysis focuses on the comparative robustness and relative error reduction achieved by the various placement strategies within a real-world, occluded environment.

### Baseline deployment strategies

The proposed strategy is compared with the following benchmark deployment paradigms:

1. *Greedy entropy selection*

To assess the efficacy of the proposed global optimization strategy, we implemented a baseline referred to as the Huang-inspired Entropy-Greedy (HIEG) selection. This benchmark is inspired by the Orthogonal Matching Pursuit (OMP) principle in sensor selection^1^ and iteratively selects markers that maximize the average reduction in differential entropy across all reference camera poses. At each iteration, the algorithm selects the marker ${m}_{i}$ that satisfies:

13 $$\arg \max_{i \in R} \frac{1}{{N_{c} }}\mathop \sum \limits_{j = 1}^{{N_{c} }} \left( {H\left( {F_{j}^{curr} } \right) - H\left( {F_{j}^{curr} + F_{i,j} } \right)} \right)$$

where $H\left(F\right)=\frac{1}{2}\mathrm{l}\mathrm{n}({\left(2\pi e\right)}^{k}\mathrm{d}\mathrm{e}\mathrm{t}{(\mathrm{F})}^{-1})$ represents the differential entropy, ${F}_{j}^{curr}$ is the accumulated Fisher Information for the *j-th* camera pose, and ${F}_{i,j}$ is the information contribution of the candidate marker.

It should be emphasized that the original OMP framework in^1^ cannot be directly applied to the present problem formulation due to fundamental differences in both the sensing model and optimization objective. The method in^1^ optimizes camera localizability through entropy reduction by jointly considering natural image features and fiducial markers within a discrete greedy placement framework. In contrast, the proposed approach formulates marker placement as a Fisher Information Matrix optimization problem derived solely from pixel-level reprojection measurements of fiducial markers. Furthermore, the information contribution of each marker in the proposed framework is strongly dependent on viewpoint visibility, line-of-sight conditions, and mesh-based occlusion, resulting in pose-dependent information matrices rather than a fixed localizability representation. Consequently, the exact OMP residual-update mechanism used in^1^ is not directly compatible with the present visibility-aware FIM formulation. Therefore, an entropy-greedy variant inspired by the iterative selection principle of OMP is adopted as a practical comparison baseline.

2. *Random placement*: Marker positions are sampled randomly across the workspace.
3. *Uniform grid placement*: Markers are evenly spaced on all visible surfaces.

### Evaluation metrics

We evaluate placement strategies across multiple quantitative metrics:

#### Reprojection error

The reprojection error measures the discrepancy between the observed 2D marker corners and the projected corners from the estimated 3D pose:

14 $$RE = \frac{1}{{N_{c} N_{i} N_{k} }}\mathop \sum \limits_{c = 1}^{{N_{c} }} \mathop \sum \limits_{{{\mathrm{i}} = 1}}^{{N_{i} }} \mathop \sum \limits_{{{\mathrm{k}} = 1}}^{4} \left\| {z_{{c,{\mathrm{i}},{\mathrm{k}}}} - h\left( {x_{{i,{\mathrm{k}}}} } \right)} \right\|_{2}$$

where $N_{c} ,{ }N_{i} ,{ }N_{k}$ = 4 is number of camera poses, markers and corners per marker respectively. $z_{c,i,k}$ is observed 2D position of corner *p* of marker *m* in camera *c*. $x_{i,k}$ is the 3D position of marker corner *p*.

This gives the average pixel error per corner over all cameras and markers.

#### Translational RMSE (cm)

15 $$RMSE_{p} = \sqrt {\frac{1}{k}\mathop \sum \limits_{k} \left\| {\hat{t}^{\left( k \right)} - t^{\left( k \right)} } \right\|^{2} }$$

where *K* denotes the total number of evaluated camera poses, ${t}^{(k)}\in {\mathbb{R}}^{3}$ represents the ground-truth translation vector for the *k*-th pose, and ${\widehat{t}}^{(k)}$ denotes the corresponding estimated translation obtained from the pose-estimation algorithm. The Euclidean norm $\parallel \cdot \parallel$ measures the translational error magnitude in three-dimensional space.

#### Rotational RMSE (deg)

16 $$RMSE_{r} = \sqrt {\frac{1}{k}\mathop \sum \limits_{k} \left\| {\hat{r}^{\left( k \right)} - r^{\left( k \right)} } \right\|^{2} }$$

where ${r}^{(k)}$ and ${\widehat{r}}^{(k)}$ denote the ground-truth and estimated rotation representations for the *k*-th pose, respectively. The rotational error is expressed in degrees and computed from the difference between the estimated and reference camera orientations over all evaluated poses.

#### Workspace coverage

The workspace coverage measures the fraction of candidate marker locations that are observable by at least one camera:

17 $$\:Coverage=\frac{1}{{N}_{i}}\sum\:_{i=1}^{{N}_{i}}\mathbbm{l}\left(\exists\:c\in\:\left[1,{N}_{c}\right]\:s.t.\:marker\:m\:is\:visible\:in\:camera\:c\right)$$

where ${\mathbbm{l}}(.)$ is indicator function (1 if true, 0 otherwise). A marker is visible if it satisfies distance, field-of-view, and occlusion constraints.

## Results

### Simulation-based quantitative evaluation

To evaluate the proposed marker-placement strategy across diverse indoor layouts, we selected three representative Matterport3D virtual environments (Scenes #1–3) and one physical laboratory testbed. The Matterport scenes were chosen to represent distinct geometric characteristics: corridor-dominant layouts, open-plan spaces, and cluttered residential environments. These varied geometries allow for a robust assessment of the algorithm under different visibility and occlusion patterns.

The physical laboratory (Scene #4) serves as a real-world validation site, featuring internal partitions and furniture that introduce practical sensing challenges not fully captured in simulation. The architectural specifications and experimental configurations for all four environments, including navigable areas and candidate camera and marker counts, are detailed in Table 1.

**Table 1:** Geometric and architectural specifications of the Matterport3D environments used for experimental evaluation.

Scene number	Matterport scene ID	Cameras	Dimension (${m}^{2}$)	Markers
#1	aayBHfsNo7d	3192	256.73	150
#2	1pXnuDYAj8r	5368	501.50	227
#3	8WUmhLawc2A	5088	518.26	422
#4	Physical Lab	52	56.06	45

Table 2 summarizes the Monte Carlo localization performance across four indoor scenes with varying geometric complexity and marker counts. The number of selected markers K was determined individually for each scene according to the spatial extent and geometric complexity of the environment, while ensuring sufficient localization coverage under visibility and occlusion constraints. In all evaluated environments, the proposed FIM-based placement strategy consistently achieved the lowest translational and rotational RMSE while maintaining the highest or near-highest localization coverage. In Scene #1, the proposed method reduced translational RMSE from 9.20 to 6.88 cm compared with the HIEG baseline, while improving coverage from 53.4 to 73.5%. Similar trends were observed in Scene #2 and Scene #3, where the proposed framework achieved translational errors below 7 cm and rotational errors close to ${1}^{^\circ }$, significantly outperforming both Uniform and Random deployments, which frequently exceeded 35 cm translational error. The results indicate that visibility-aware FIM optimization produces substantially more informative and spatially balanced marker configurations than heuristic placement approaches.

**Table 2:** Comparison of pose estimation accuracy and spatial coverage across different marker placement strategies.

Scene ID/method	Trans. RMSE ($\mathrm{c}\mathrm{m}$)	Rot.RMSE (°)	Reproj. (mrad)	Coverage (%)	K
Scene #1					
FIM	6.88 ± 0.80	1.16 ± 0.08	0.19 ± 0.01	73.5 ± 0.0	20
HIEG	9.20 ± 0.94	1.48 ± 0.11	0.24 ± 0.01	53.4 ± 0.0	
Uniform	52.64 ± 5.31	4.72 ± 0.49	0.20 ± 0.01	34.3 ± 0.0	
Random	48.07 ± 6.07	4.45 ± 0.48	0.20 ± 0.01	49.6 ± 0.0	
Scene #2					
FIM	6.54 ± 0.00	1.02 ± 0.00	0.18 ± 0.00	74.4 ± 0.0	29
HIEG	10.04 ± 0.00	1.58 ± 0.00	0.22 ± 0.00	62.1 ± 0.0	
Uniform	37.70 ± 0.00	3.91 ± 0.00	0.22 ± 0.00	29.0 ± 0.0	
Random	39.45 ± 0.00	3.85 ± 0.00	0.29 ± 0.00	28.3 ± 0.0	
Scene #3					
FIM	4.89 ± 0.19	1.05 ± 0.04	0.26 ± 0.01	77.9 ± 0.0	38
HIEG	8.88 ± 0.11	1.40 ± 0.01	0.27 ± 0.01	70.7 ± 0.0	
Uniform	45.85 ± 0.94	4.39 ± 0.17	0.29 ± 0.02	29.6 ± 0.0	
Random	39.75 ± 10.61	3.95 ± 1.05	0.27 ± 0.00	28.8 ± 0.0	
Scene #4					
FIM	4.03 ± 0.10	1.50 ± 0.03	0.89 ± 0.02	75.5 ± 0.0	16
HIEG	4.66 ± 0.58	1.57 ± 0.12	0.91 ± 0.02	73.1 ± 0.0	
Uniform	6.09 ± 0.31	1.90 ± 0.07	0.92 ± 0.02	51.9 ± 0.0	
Random	5.07 ± 0.75	1.57 ± 0.16	0.69 ± 0.03	50.0 ± 0.0	

In Scene #4, where the environment contains fewer feasible marker locations and lower geometric diversity, the performance gap between the proposed method and the entropy-based baseline became smaller; nevertheless, the proposed framework still achieved the best overall localization accuracy with 4.03 cm translational RMSE and 1.50° rotational RMSE. Across all scenes, Uniform and Random placements exhibited notably lower coverage and substantially larger pose estimation errors, demonstrating that naive spatial distribution alone does not guarantee robust localization performance. Overall, the results validate that explicitly incorporating Fisher-information maximization together with visibility and occlusion constraints leads to consistently improved pose estimation accuracy and environmental coverage in realistic indoor environments.

**Fig. 3:**
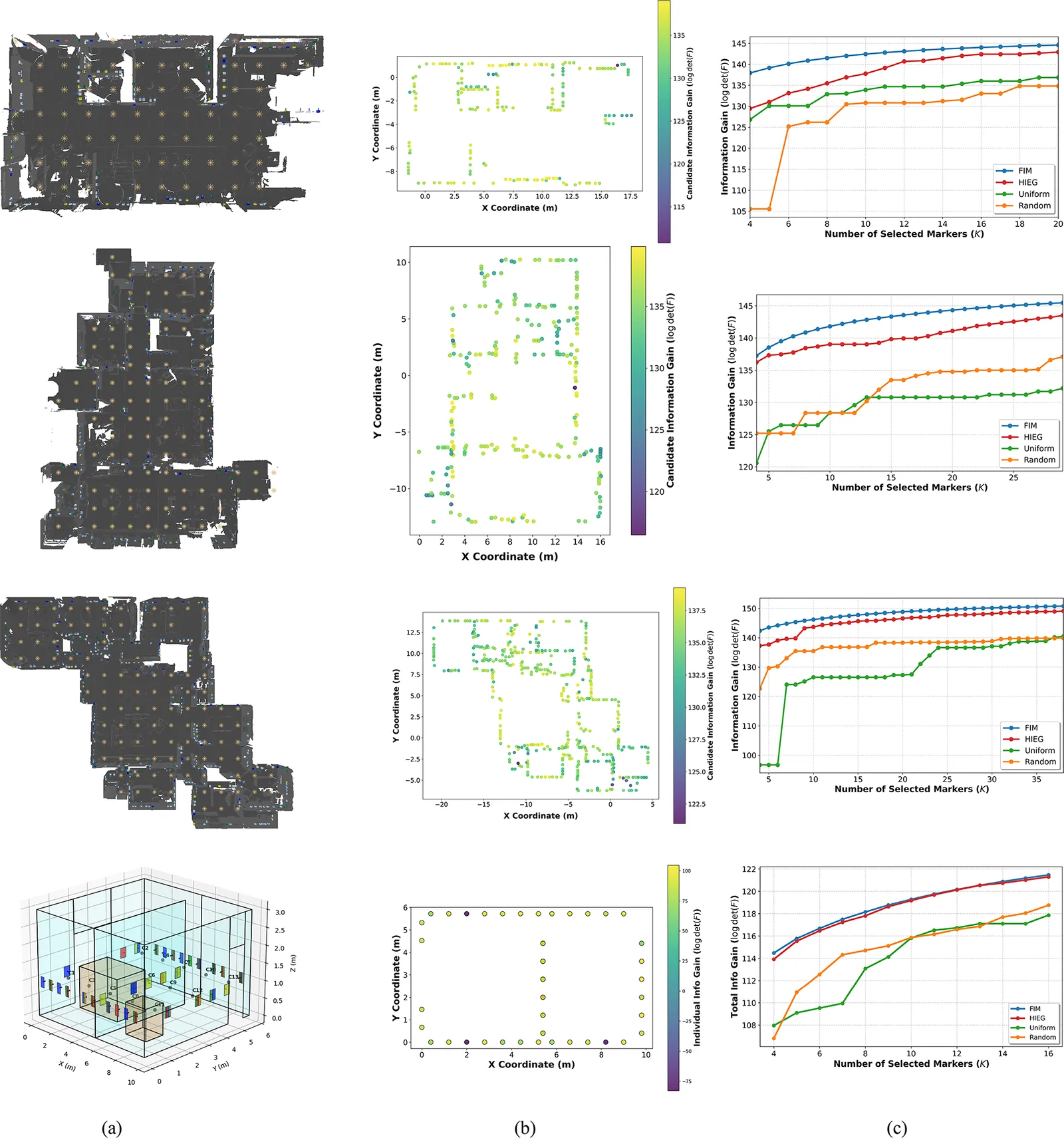
Qualitative analysis of the marker optimization process across three test environments. (**a**) Spatial distribution of candidate and selected markers where marker colors denote placement strategies: blue—FIM-optimized, red—HIEG baseline, yellow—uniform, green—random, and white–gray—candidate locations. (**b**) Log-determinant Fisher Information heatmaps (log det(F)) representing surface-level geometric observability. (**c**) Information gain curves illustrating the cumulative log-determinant as a function of the number of selected markers (k), highlighting the selection efficiency of the greedy FIM-driven strategy.

To provide a deeper understanding of the proposed framework, the qualitative visual comparisons for each scene demonstrate the spatial logic of the optimization process. The visual analysis is divided into three primary components: the environment layout with candidate and selected poses, the Fisher Information heatmap, and the information gain curves (Fig. 3). In the environment visualization, all feasible candidate marker positions are indicated in white, while the markers selected by the different strategies are highlighted for comparison. The proposed FIM-optimized placement is shown in blue, the HIEG baseline in red, the uniform grid selection in yellow, and the random selection in green.

The wall-surface information heatmaps, illustrated in Fig. 3b, which represent the log-determinant of the Fisher Information Matrix $\mathrm{log}\mathrm{d}\mathrm{e}\mathrm{t}(F)$ reveal that the highest information density typically clusters around critical architectural features such as corners, edges, and long, unobstructed wall sections. The FIM-based selection strategy closely tracks these high-information regions, ensuring that the markers are placed where they contribute most to the geometric stability of the pose estimate.

### Sensitivity and robustness analysis

To evaluate the robustness of the proposed framework, we conducted a comprehensive sensitivity analysis across four critical dimensions: sensor noise levels, marker physical dimensions, information gain stability, and calibration deviations.

#### Sensor noise and marker scaling

**Fig. 4:**
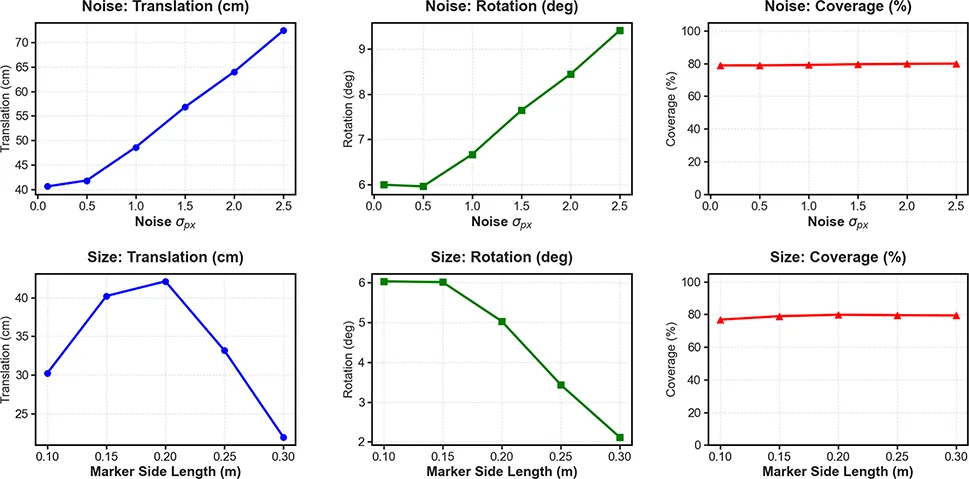
Sensitivity analysis of the proposed FIM-based marker placement strategy across varying operational parameters.

The system’s performance under varying sensor noise *σ* is illustrated in the top row of Fig. 4. As expected, translational and rotational RMSE exhibit a nearly linear correlation with increasing noise, yet remain within operational bounds. Notably, the Coverage (%) remains consistently high, suggesting that the FIM-based selection prioritizes “geometrically redundant” placements that maintain visibility despite noisy corner detections.

The impact of Marker Side Length (Fig. 4, bottom row) reveals an optimization peak. At small scales, a poor signal-to-noise ratio leads to higher RMSE, while increasing the side length provides a significant performance gain until reaching a saturation point. Specifically, the rotational error shows a distinct downward trend with larger markers, confirming they provide a more stable baseline for orientation estimation.

#### Information gain consistency

**Fig. 5:**
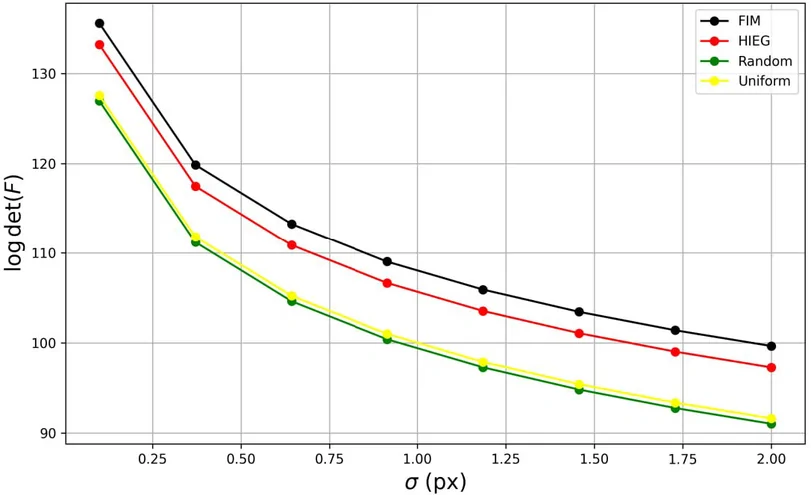
Sensitivity of information gain to measurement noise.

As depicted in Fig. 5, the FIM-optimized configurations maintain a consistent information margin over uniform and random strategies across the entire noise spectrum, from 0.2 to 2.0 pixels. This stable offset confirms that the performance advantage of the proposed method is a fundamental property of its geometry-aware selection process rather than a byproduct of ideal, low-noise conditions.

#### Calibration robustness

**Fig. 6:**
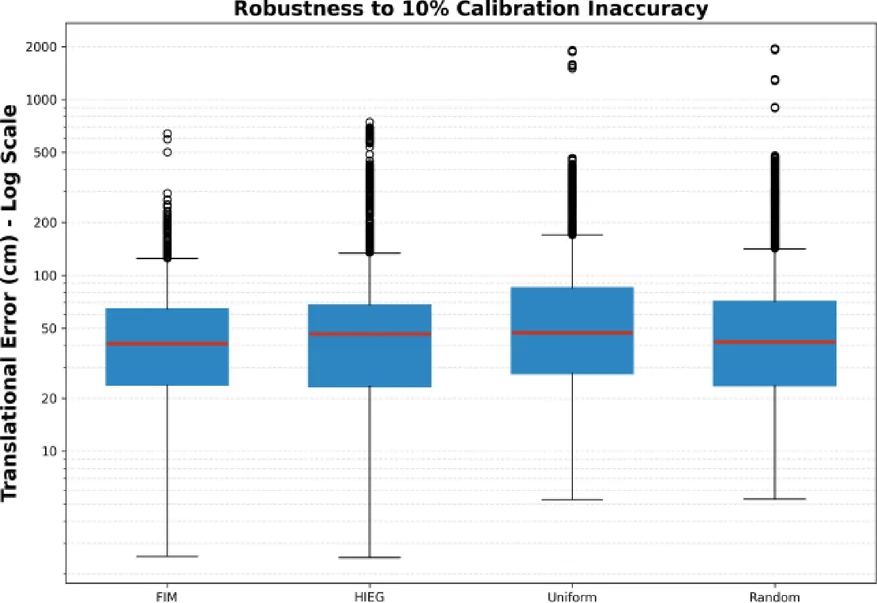
Robustness to calibration inaccuracies. The boxplot illustrates the translational error distribution under a 10% deviation in camera intrinsic parameters.

To assess real-world applicability, we introduced a 10% calibration deviation to simulate intrinsic parameter inaccuracies. In the error distribution shown in Fig. 6, the FIM-optimized strategy exhibits a lower median error and reduced variance compared to other baselines, highlighting its superior resilience to modeling uncertainties. While all methods experience increased variance, the FIM-optimized configurations demonstrate tighter error distributions and fewer extreme outliers compared to the uniform and random baselines. This indicates that FIM-guided placement results in a more well-conditioned system that is less sensitive to modeling errors.

### Experimental validation in physical environment

**Fig. 7:**
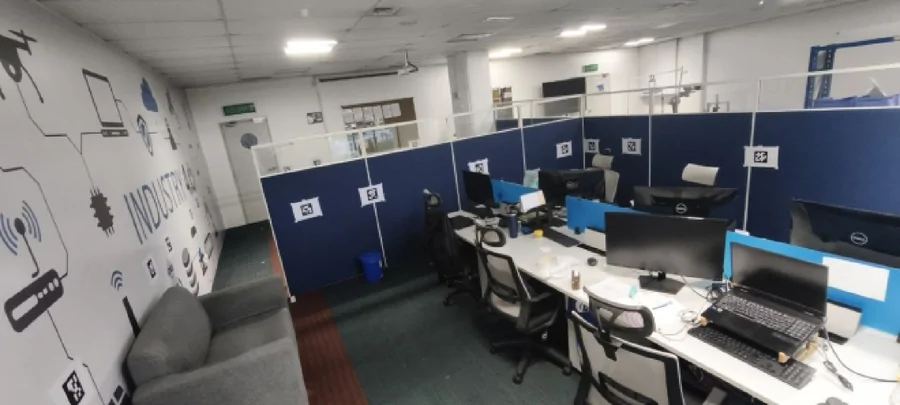
Physical experimental environment.

To bridge the gap between simulation and real-world application, the optimized configurations were deployed in the physical laboratory testbed, as illustrated in Fig. 7. The hardware specifications, including the Teledyne FLIR camera intrinsics and distortion coefficients, are expressed in Section “Physical laboratory testbed”. For each deployment configuration, camera poses were estimated using detected AprilTag observations and compared against manually measured reference poses at predefined test locations.

**Fig. 8:**
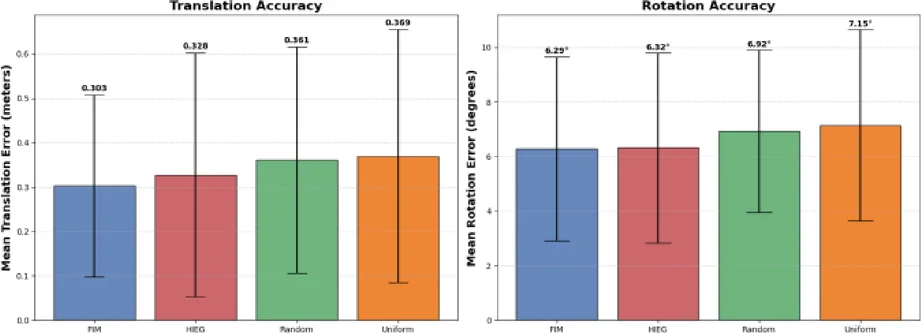
Comparative pose estimation performance in the physical lab.

Figure 8 summarizes the experimental localization performance for the evaluated deployment strategies. As expected, the real-world errors are higher than the simulation results due to practical factors including calibration uncertainty, image noise, illumination variations, imperfect marker detection, and inaccuracies in manual ground-truth measurements. In particular, the manually measured reference poses introduce unavoidable translational and rotational uncertainty that cannot be completely eliminated in a laboratory-scale experiment.

Nevertheless, the relative performance trends remained consistent with the simulation study. The proposed visibility-aware FIM optimization achieved the lowest overall translational and rotational error, while the HIEG baseline provided intermediate performance and Random deployment produced the largest localization errors. These results experimentally validate that maximizing Fisher Information under realistic visibility and occlusion constraints leads to more reliable pose estimation performance in real indoor environments.

## Conclusion

This paper presented a visibility-aware Fisher Information Matrix (FIM) optimization framework for optimal fiducial marker deployment in complex indoor environments. Unlike conventional heuristic, uniform, or visibility-only placement strategies, the proposed method formulates marker deployment directly from pixel-level reprojection uncertainty while explicitly incorporating environmental visibility and occlusion constraints. By integrating geometric observability with realistic line-of-sight accessibility, the framework provides a physically grounded and statistically principled criterion for marker placement.

The proposed method was validated through both simulation and real-world experiments conducted in a laboratory-scale indoor environment. Simulation results demonstrated that the proposed visibility-aware FIM optimization consistently achieved lower translational and rotational pose-estimation errors compared with entropy-based Huang-inspired optimization, uniform placement, and random deployment strategies. Experimental validation further confirmed the practical effectiveness of the approach under realistic sensing conditions, where the proposed deployment achieved improved localization accuracy and robustness despite measurement noise, occlusions, and hand-measured ground-truth uncertainties.

### Limitations and future work

Despite the promising results, several limitations should be acknowledged. First, the proposed framework assumes Gaussian pixel-level measurement noise to enable tractable FIM analysis; however, this assumption is commonly adopted in estimation-theoretic localization and calibration frameworks, practical fiducial detection systems may exhibit non-Gaussian, correlated, or image-dependent errors caused by illumination variation, motion blur, partial occlusion, lens distortion, and detector quantization effects. Consequently, the current formulation may not fully capture the statistical characteristics of real-world marker detection uncertainty under challenging sensing conditions. Nevertheless, the Gaussian approximation provides a computationally efficient and analytically interpretable basis for evaluating geometric observability and relative deployment quality. Future work will investigate robust and heteroscedastic noise models for visibility-aware marker placement under realistic detection uncertainty. Second, the current formulation assumes a static environment and does not explicitly model dynamic obstacles or time-varying occlusions that may affect marker visibility in practical deployments. Third, although real-world experiments were conducted, the evaluation remains limited to a laboratory-scale environment with manually measured ground-truth poses, which introduces unavoidable reference uncertainty. Finally, the greedy optimization strategy provides computational efficiency but does not guarantee global optimality. Future work will investigate robust noise modeling, dynamic visibility-aware deployment, larger-scale experimental validation, and global optimization strategies for adaptive marker placement.

## Data Availability

The datasets analyzed during the current study were obtained from the Matterport3D group. Access to these data can be requested by contacting the group administrators at matterport3d@googlegroups.com. The authors do not have the authority to redistribute this third-party data.

## Appendix

Jacobian for one corner: let the corner point in world *p*, Camera poses $\theta =(t,\phi )$ so that camera rotates points via *R(ϕ )* and translates by *t*. The projection is *z=h(R(p-t))* Using chain rule:

18 $$\frac{\partial \left( p \right)}{{\partial t}} = - R$$

19 $$\frac{\partial \left( p \right)}{{\partial \phi }} = - R\left[ q \right]_{ \times }$$

20 $$\left[ q \right]_{ \times } = \left[ {\begin{array}{*{20}c} 0 & { - q_{z} } & {q_{y} } \\ {q_{z} } & 0 & { - q_{x} } \\ { - q_{y} } & {q_{x} } & 0 \\ \end{array} } \right]$$

where *q = p - t*, denotes the skew-symmetric matrix. The perspective projection Jacobian for a single marker corner observation is given by

21 $$\frac{{\partial \left( {u,v} \right)}}{\partial \left( p \right)} = \left[ {\begin{array}{*{20}c} {\begin{array}{*{20}c} {\frac{{f_{x} }}{Z}} \\ 0 \\ \end{array} } & {\begin{array}{*{20}c} 0 \\ {\frac{{f_{y} }}{Z}} \\ \end{array} } & {\begin{array}{*{20}c} {\frac{{ - f_{x} X}}{{Z^{2} }}} \\ {\frac{{ - f_{y} Y}}{{Z^{2} }}} \\ \end{array} } \\ \end{array} } \right]$$

Applying the chain rule, the complete pose Jacobian for one marker corner observation becomes

22 $$J = \frac{\partial h\left( p \right)}{{\partial \left( p \right)}}\frac{\partial \left( p \right)}{{\partial \left[ {\phi ,t} \right]}}$$

23 $$\begin{aligned} J = & \frac{{\partial \left( {u,v} \right)}}{\partial \left( p \right)}\left[ {\begin{array}{*{20}c} { - R\left[ q \right]_{ \times } } & { - R} \\ \end{array} } \right] \\ = & \left[ {\begin{array}{*{20}c} {\begin{array}{*{20}c} {\frac{{f_{x} }}{Z}} \\ 0 \\ \end{array} } & {\begin{array}{*{20}c} 0 \\ {\frac{{f_{y} }}{Z}} \\ \end{array} } & {\begin{array}{*{20}c} {\frac{{ - f_{x} X}}{{Z^{2} }}} \\ {\frac{{ - f_{y} Y}}{{Z^{2} }}} \\ \end{array} } \\ \end{array} } \right]\left[ {\begin{array}{*{20}c} { - R\left[ q \right]_{ \times } } & { - R} \\ \end{array} } \right] \\ \end{aligned}$$

## Code availability

The custom code developed for the simulations and experimental validation in this study is available from the corresponding authors upon reasonable request.
